# Development and Validation of an Acute Large Animal Model for Type A Aortic Dissection

**DOI:** 10.3390/jcdd12120496

**Published:** 2025-12-16

**Authors:** Ezin Deniz, Sibylle Marsen, Florian Helms, Heike Krüger, Naoki Arima, Jasmin Hanke, Ali Saad Merzah, Sadeq Al-Hasan-Al-Saegh, Sara Knigge, Saman Alhowaizy, Tanja Meyer, Rabea Hinkel, Morsi Arar, Aron F. Popov, Günes Dogan, Bastian Schmack, Alexander Weymann, Arjang Ruhparwar, Salaheldien Ali Mohamed-Glüer, Jan D. Schmitto

**Affiliations:** 1 Department of Cardiac, Thoracic, Transplantation and Vascular Surgery, Hannover Medical School, 30625 Hannover, Germany; 2 Pericardcheck GmbH, 28211 Bremen, Germany; 3 Medimplant GmbH, 30173 Hannover, Germany; 4 Institute for Animal Hygiene, Animal Welfare and Farm Animal Ethology (ITTN), University of Veterinary Medicine Hannover, 30559 Hannover, Germany; 5 Laboratory Animal Science Unit, German Primate Center (DPZ), Leibniz Institute of Primate Research, 37077 Göttingen, Germany; 6 German Center for Cardiovascular Research (DZHK), Partner Site Lower Saxony, 37077 Göttingen, Germany; 7 Department of Cardiac Surgery, Asklepios Klinikum Harburg, 21075 Hamburg, Germany; 8 University Hospital Schleswig-Holstein, UKSH Campus Luebeck, 23538 Luebeck, Germany

**Keywords:** aortic dissection, type A, large animal model

## Abstract

Background: Animal models are essential for translating diagnostic and therapeutic strategies into clinical practice and offer valuable insights into the pathophysiology of diseases such as aortic dissection. This study presents a novel acute in vivo large animal model of Stanford type A aortic dissection, combining open surgical access with endovascular techniques to leverage the advantages of both. The model aims to reproducibly simulate acute dissections in swine, providing a standardized platform for evaluating diagnostics, disease mechanisms, and treatment strategies. Methods: Six pigs underwent a standardized protocol to induce aortic dissection. Arterial pressure was monitored via femoral and carotid catheterization. A conventional sternotomy was performed, followed by tangential cross-clamping of the ascending aorta and a controlled incision proximal to the brachiocephalic trunk. The intima and the media were separated using a guidewire and catheter-based technique to create a false lumen. A re-entry tear was also established to allow for controlled intraluminal access. Animals were monitored for 12 h post-intervention, with serial blood sampling. At the end of the experiment, the animals were euthanized and the aortas harvested for macroscopic and histological analysis. Results: In all 6 animals, the placement of arterial catheters in femoral and carotid arteries, as well as the sternotomy, was established without any complications. The dissection model was successfully created in 5 out of 6 animals by clinical signs such as adventitial hematoma, macroscopic wall separation and/or decreased femoral blood pressure. One animal experienced complete aortic perforation. Five animals completed the full observation period of 12 h. Conclusion: A standardized, reproducible, and robust large animal model of acute Stanford type A aortic dissection using a hybrid approach was developed. This model closely simulates the clinical and pathological features of human aortic dissection, making it a valuable tool for preclinical research in diagnostics, pathophysiology, and treatment development.

## 1. Introduction

Despite progress in diagnosis, optimal medical management, and advances in surgical techniques, the in-hospital mortality rate of type A aortic dissection remains as high as 26%, with prehospital mortality also significant [1]. Moreover, acute type A aortic dissections, which involve the ascending aorta according to the Stanford classification, are life-threatening medical emergencies with mortality increasing up to 1% per hour in the first 48 h [2,3,4]. In the International Registry of Acute Aortic Dissections, 62.3% of the total of 464 patients had type-A dissections [1].

Therefore, establishing reproducible large animal models of acute Type A aortic dissections is essential, particularly in pigs due to their anatomical similarity to humans. In 1959, Blanton et al. [5] first established an aortic dissection model using a surgical method. With the advances in interventional techniques, several in vivo and ex vivo experimental models of aortic dissection have previously been described [5,6,7,8,9,10,11,12,13]. However, only a few type A aortic dissection models in large animals have been reported [7,8,11] in the past several decades. Moreover, there is no animal model combining endovascular and surgical techniques to create a type A dissection [14,15,16,17].

These models were performed with a surgical procedure based on the modified Blanton’s technique, requiring thoracotomy or sternotomy and cross-clamping of the ascending aorta. Due to their invasive nature, these approaches are associated with high animal mortality and limited reproducibility. On the other hand, endovascular procedures are less invasive and more feasible than previously reported surgical models; therefore may be more applicable and reproducible [12]. The endovascular model is closer in its conception to the pathogenesis of spontaneous aortic dissection in humans, as it is primarily based on the creation of an intimal tear from within the vessel, whereas in the surgical approach, the access to create the intimal tear is necessarily via the adventitia of the aortic wall. The dissection is created under direct vision during the surgical procedure, but the extent of the dissection is limited.

This study describes the first in vivo model of acute type A aortic dissection using a combined surgical and catheter-based approach, designed to enable scientific investigation of the disease. As the model was conceived as an acute model, the criteria retained for its validity were that it should be reproducible, nonlethal, and stable during the procedure.

## 2. Methods

### 2.1. Animals

This study was approved by the Niedersächsische Landesamt für Verbraucherschutz und Lebensmittelsicherheit (LAVES) (33.19-42502-04-24-00728). It was conducted by an interdisciplinary heart team. The animals chosen were pigs, mainly because of their generally accepted anatomical and hemodynamic similarities to humans. All experiments were performed in accordance with the guidelines of the Lower Saxony State Office for Consumer Protection and Food Safety (LAVES), Germany. Six swine (2 female, 4 male; age 7 months; mean weight 69.7 kg, range 60–83 kg) were used in this study. Animals were preanaesthetized with azaperone (2 mg/kg, i.m.) and ketamine (50 mg/kg, i.m.), followed by general anesthesia with isoflurane (2–3 Vol.%) in the supine position with continuous monitoring. Blood samples were taken according to a standardized protocol at baseline before the procedure, as well as intraoperatively according to the protocol. Procedures were carried out with the localization of dissection in the ascending aorta. Animals were followed up for 12 h to evaluate the hemodynamic, anatomical, and laboratory conditions. At the end of the experiment, the sedated animals were euthanized with 40 mEq of intravenous potassium chloride. Blood samples were collected at baseline and every hour after the procedure.

The technical success criteria of our animal model were derived from this pathophysiological definition to include the creation of an intimal tear in the ascending aorta and a double lumen aorta, whether dissection extended to the descending thoracic aorta or not, and whether a re-entry tear was created or not. It was assessed after the procedure by the false lumen by macroscopic and histological confirmation. Neither computed tomography (CT) imaging, angiography, nor duplex scan was used for this evaluation.

### 2.2. Study Design

Ascending aorta dissection entry was performed in all 6 pigs. In two pigs as a control group, the aorta ascendens were explanted non-manipulated (Table 1). Blood samples and hemodynamic parameters were collected at each of the following time points: before thoracotomy but after induction of anesthesia as baseline (T1), immediately after the dissection (T2), and every hour after dissection for 12 h (T3–T14). Venous blood was collected from the central venous catheter. All of the pigs were euthanized at T14 (12 h after the surgery) for the collection of pathological specimens. The whole aorta and heart were resected en bloc, and the aortic dissection was evaluated to measure the tear length.

### 2.3. Surgical and Endovascular Procedure

After routine disinfection and draping, two arterial catheters and one central venous catheter were placed. Arterial access was established via surgical cut-down and puncture of the left common femoral artery, and via percutaneous puncture of the right common carotid artery. These access sites allowed continuous monitoring of arterial pressure in both cranial and caudal directions, which was essential for clinical evaluation of the dissection.

Then, a median sternotomy was performed to expose the ascending aorta, and the pericardium was suspended. Creation of entry tear was in all cases achieved by instrumental means, with tangentianial crossclamping of the aorta, then incision of the ascending aorta, splitting the walls with infusion of NaCl, placing a guide needle and a guidewire into the descending aorta. Aortic dissection was established in the ascending aorta.

### 2.4. Data Analysis

The data are presented as the mean ± SD. The paired *t*-test was used for comparisons among groups, and the pairwise *t*-test was used for comparisons within groups. A significance level of α = 0.05 was selected, and *p* < 0.05 was considered statistically significant.

## 3. Results

### 3.1. Technical Results

All six animals tolerated the surgical procedures without major intraoperative complications. Femoral and carotid arterial catheter placement was achieved in all cases, allowing for real-time monitoring of central and peripheral arterial pressures. Standard median sternotomy and pericardial suspension provided adequate exposure of the ascending aorta in each animal. Surgical instrumentation and hemodynamic monitoring were performed under stable conditions with minimal blood loss and no major arrhythmias.

**Figure 1:**
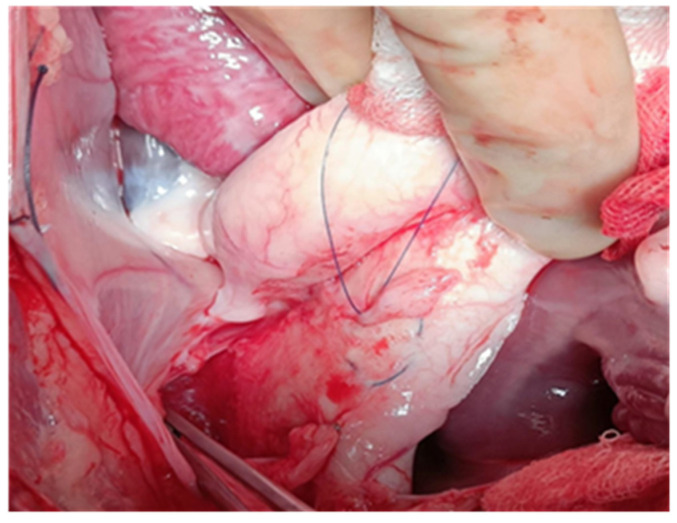
Tobacco-pouch in the ascending aorta.

In all cases, at about 1 cm proximal from the initiation site of the brachiocephalic trunk, one tobacco-pouch suture was placed, and the aortic wall was clamped in this area longitudinally. The aorta was cut between the clamp in the area of the tobacco-pouch suture in a length of 1 cm. A small round blade was used to incise through the adventitia and part of the media 1 cm in length (Figure 1).

**Figure 2:**
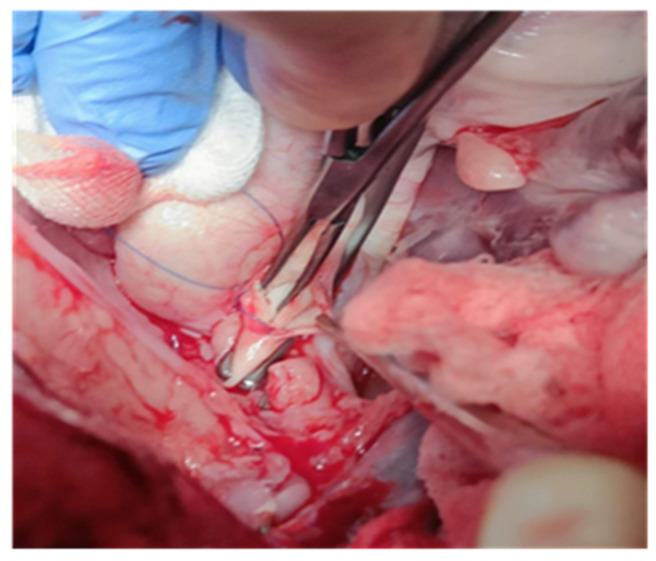
Cross-clamped the aorta and incision proximal to the bracheocephalic trunk.

The media and the intima were incised and communicated with the aortic cavity, then a fan-shaped part of the media and the intima of the distal incision were removed. After exposing the medial space, the intima was split from the media with a dissector. The tip of a mosquito clamp and the dissector were used for blunt, gentle dissection along the medial space in the distal direction between the clamp branches, which was lengthened about 1 cm (Figure 2).

**Figure 3:**
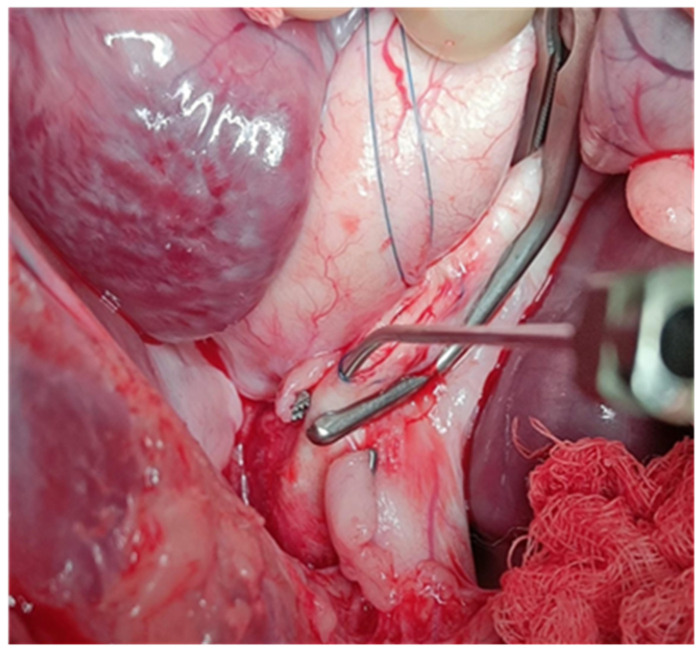
Splitting the aortic wall layers by NaCl infusion via a cannula.

In this space, a cannula was first inserted, and 20 mL of NaCl solution was injected between the layers of the aortic walls to split the walls. Then the cannula was pushed approximately 2 cm between the walls through this access and reinserted into the true lumen in the arch until blood flushing (Figure 3).

**Figure 4:**
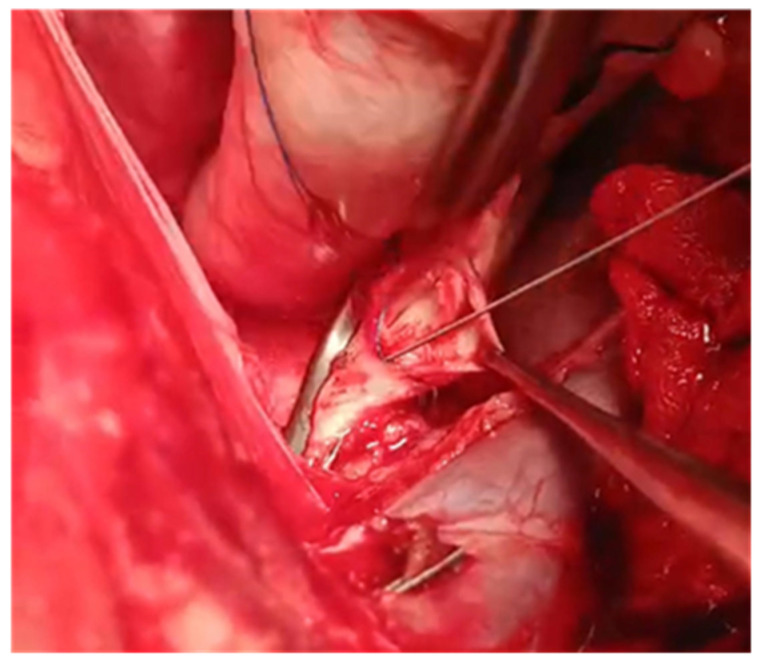
Extended the aortic dissection using a guidewire and balloon catheter.

A guidewire was inserted into the aorta by using the Seldinger technique, after which the cannula was removed. A balloon catheter was then introduced over the guidewire. The balloon was inflated and gradually pulled back at 1 cm intervals to expand the dissection area under high pressure (Figure 4). This process was continued until the dissection extended about 2 cm into the aortic arch.

**Figure 5:**
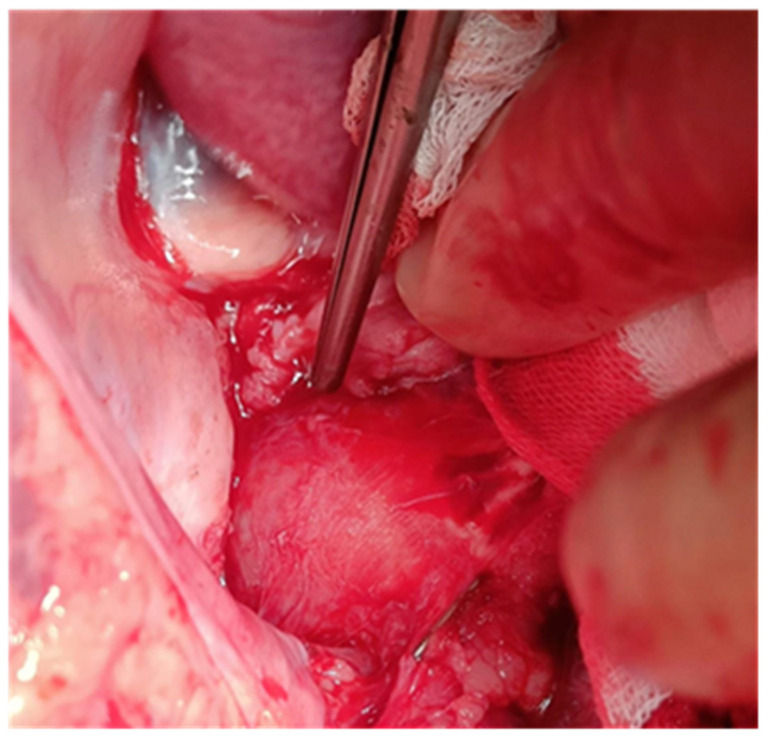
Macroscopic assessment of hematoma formation.

After the balloon catheter was removed, the aortic incision was closed using a tobacco-pouch suture. The guidewire was secured in place during the closure. The aortic clamp was then released, and a second layer of sutures was reinforced. Finally, the incision site was examined macroscopically to assess for hematoma formation (Figure 5).

In all cases, after initial insertion, the aortic wall was punctured using either of the previously mentioned devices, and a guidewire was advanced approximately 2 cm into the wall through a cannula. A cannula, a guidewire, and a 4-F angiographic catheter were then advanced into the entry tear. The dissection was further extended in the antegrade direction by pushing forward the looped guidewire, supported by a straight catheter. Re-entry into the true lumen was achieved by forcefully pushing the cannula into the true lumen until blood flushing. The hybrid dissection technique was effective in five of the six animals. In these cases, dissection was confirmed through a combination of clinical indicators, including macroscopic wall separation, adventitial hematoma formation, and a decrease in distal (femoral) arterial pressure. Although femoral arterial pressure tended to decrease following successful dissection induction, the observed changes did not reach statistical significance due to interindividual variability and the limited sample size. Therefore, quantitative analysis of pressure gradients was not included in the present results and served primarily as a qualitative intraoperative indicator of reduced distal perfusion.

One animal suffered from an iatrogenic full-thickness aortic wall perforation during the iatrogenic onset of the dissection and was euthanized immediately due to hemodynamic collapse. This case was excluded from follow-up assessment, and after euthanasia, the aorta was explanted for pathological evaluation.

**Figure 6:**
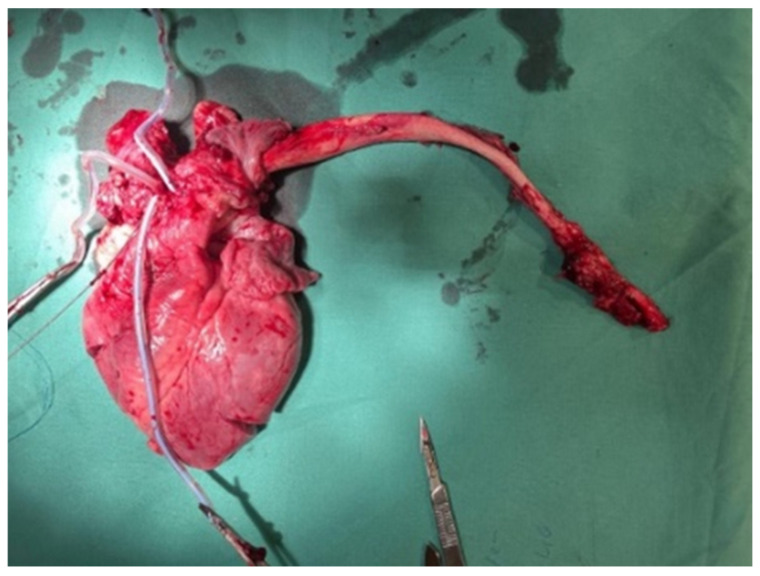
En bloc resection of heart and thoraco-abdominal aorta.

A CT scanner was not available in our laboratory to increase the documentation of the experiment even more. During the procedure and in the 12 h follow-up, blood samples were taken, and hemodynamic parameters were recorded in 5 of the 6 cases. For macroscopic and histologic examination, animals were euthanized. The heart and thoraco-abdominal aorta were then exposed en bloc in all 6 cases (Figure 6).

### 3.2. Macroscopic Results

The explanted aorta underwent pathological examination. The aorta was longitudinally opened on the opposite side to the false lumen, from the distal aorta right up to the aortic cusps. Macroscopic examination of the excised aorta was performed, and associated with caliper measurement of the following parameters: distance between the aortic cusps and the entry tear, length of the dissected aorta, and full thickness of the true lumen wall. The specimen was fixed in a 4% formalin solution, and 5-μm cross-sections were obtained at three levels: the entry tear, the dissected space, and the reentry tear when existing, and then embedded in paraffin. Afterward, serial 5-μm-thick transversal slices were prepared, stained with hematoxylin and eosin, and examined using an optical microscope.

All explanted aortic specimens (n = 8; 6 intervention pigs, 2 control pigs) were subjected to detailed macroscopic, histologic, and semiquantitative pathological examinations (Table 2). In a few cases, additional digital microscopic examinations were performed (+).

Six specimens were derived from animals with iatrogenically induced aortic dissection, while two specimens served as non-manipulated controls. Pathological analyses were conducted at the Private Institute for Implant Pathology (Private Institute for Implant Pathology, Selfkant, Germany), utilizing hematoxylin-eosin (HE), Elastica-van Gieson (EvG), and Ladewig stains to visualize vessel wall integrity, elastic fibers, muscle layers, fibrotic remodeling, and the presence of thrombotic material.

**Figure 7:**
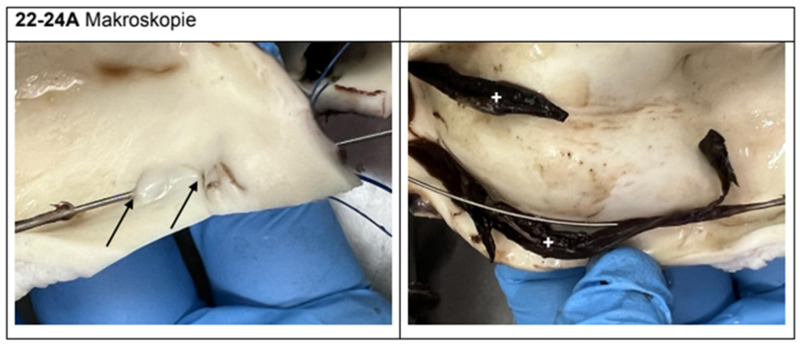
Wire course between the walls 1 cm (arrow), thrombotic material (+).

**Figure 8:**
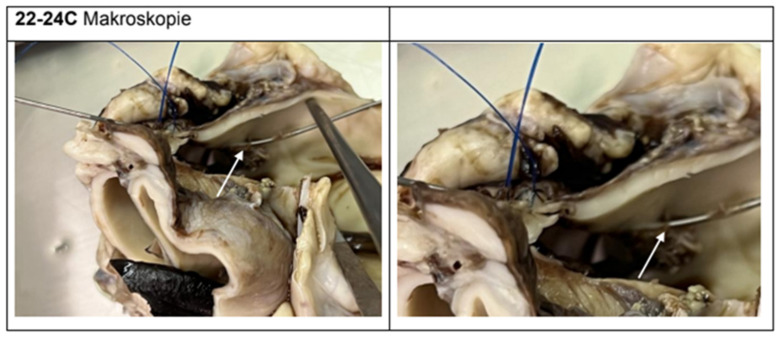
Wire course between the walls in the ascendens 1 cm (arrow).

**Figure 9:**
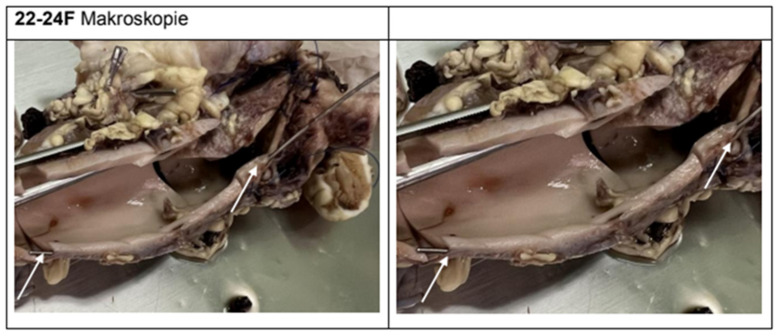
Wire course between the walls in the ascending to aortic arch 5.8 cm (arrow).

Macroscopically, five of the six experimental animals showed clear evidence of iatrogenic injury simulating aortic dissection. The intramural or transmural positioning of the wire could be traced in all relevant segments (Figure 7, Figure 8 and Figure 9). Findings included intimal tears, intramural hematomas, segmental wall bulgings, and vessel wall discontinuity. Thrombotic material was frequently observed lining the dissection planes, typically consistent with acute-phase deposition (e.g., animal 22/24 A/C/F). In contrast, the control specimens (e.g., animal 1.1/24) showed no signs of dissection, hematomas, or thrombosis. The dissection extended distally toward the aortic arch in three cases and remained confined to the ascending segment in two. Dissection lengths ranged from 1.0 cm to 5.8 cm, with a mean of 2.6 cm.

### 3.3. Microscopic Results

Histological analysis confirmed the presence of intimal disruption, separation of the media, and thrombus formation within the false lumen in all five successfully dissected specimens (Table 3). EvG and Ladewig stains revealed pronounced fragmentation of elastic fibers, loss of smooth muscle integrity, and intramural haemorrhage (Figures S1–S4). The severity of wall injury and extent of dissection were semi-quantitatively scored (Table S1). Score ranged from (+) for minimal expression to (+++++) for extremely pronounced/extensive expression. Digital microscopy further substantiated these findings in specimens with subtle morphological alterations.

In detail, following histologically, the hallmark features of acute aortic dissection were confirmed (Figures S1–S4):

- In 22-24A2 (animal 1/24), the media and adventitia exhibited a pronounced tear with disruption of elastic fiber architecture, focal hemorrhage, and fibrin deposits. Elastic fibers were shortened, rolled, or fragmented (Figure S1).
- In 22-24C4–C6 (animal 3/24), focal dehiscence and rupture of the intima and adjacent media were observed, accompanied by thrombus formation (notably in C6). Degenerative fiber loss and elastolysis were present in C5 (Figure S2).
- In 22-24D (animal 4/24), para-aortic hematomas and periadventitial hemorrhages were prominent, especially in D2–D3. Section D3 showed transmural rupture including intima, media, and adventitia (Figure S3).
- In 22-24F2 (animal 6/24), a wide, longitudinal cleavage within the media with accompanying intramural hemorrhage and thrombus formation was observed. Media fibers appeared disrupted, fragmented, and rolled (Figure S4).

Para-aortic hemorrhages and transmural disruptions were most pronounced in specimens 22-24D and 22-24F, where re-entry sites were also identified. Thrombotic material was present in both dissected and adjacent undissected zones, suggesting acute-phase vascular activation. Control specimens (22-24M and 22-24N) showed preserved vessel wall architecture without any evidence of pathology (Figures S1–S4).

Semiquantitative histological analysis of the explanted aortic specimens confirmed the presence, extent, and severity of vessel wall injury in the experimental group, whereas control specimens exhibited no relevant pathological changes (Table S1).

In specimen 22-24A2 (animal 1/24), an intimal tear was identified and scored as mild (+), while the medial tear was extensive (+++++), accompanied by a moderate adventitial tear (++). There was mild vessel wall hemorrhage (+), and the elastic fibers appeared extensively shortened, rolled, and ruptured (+++++). Muscle fibers also showed marked structural damage, being shortened and ruptured (+++++). Fibrin deposits were detected in the vessel wall, but no para-aortic hemorrhage was observed. Microscopically, this specimen showed a relatively intact wall with focal lesions extending through the media into the adventitia. Fiber bundles were interrupted and rolled at the edges, and luminal tears were associated with localized hemorrhage and fibrin deposition.

In specimen 22-24C4–6 (animal 3/24), there was a mild intimal tear (+) and a mild medial tear (+), without adventitial involvement. Vessel wall hemorrhage ranged from absent to mild ((+) ±). Elastic and muscle fiber rupture was focal and primarily located in section C5. Thrombus formation was evident in section C6 (++), while para-aortic hemorrhage was absent. Digital microscopy revealed that specimen C4 was largely intact, with only minor preparation artifacts. C5 showed focal hemorrhage with fiber dehiscence and a tear through the intima and adjacent media. In C6, thrombus formation consistent with early-stage clotting was observed.

Specimen 22-24D (animal 4/24) showed no evident intimal tear, but moderate medial (++) and adventitial (++) tearing. Vessel wall hemorrhage was also moderate (++). Elastic and muscle fibers were both severely disrupted and dehiscent (each scored as +++++), with accumulation of torn fiber fragments. Thrombus was present in several sections (+++), particularly in D1 and D3–D6. Para-aortic hemorrhage was prominent (+++), and fibrin formation was observed. Histological analysis showed thrombi in the aortic lumen, para-aortic hematomas, and, in some areas, transmural rupture. Specimens D1 and D2 indicated coagulation-type thrombus, whereas sections D3–D6 revealed thrombi of mixed types. Sections D2 and D3 also showed media tears with fiber accumulation and fibrin.

In specimen 22-24F (animal 6/24), the intimal tear was pronounced (+++), and the medial tear was scored as moderate (++), while adventitial damage was not explicitly noted. Elastic fibers were rolled, fragmented, and ruptured, and muscle fibers were shortened and similarly damaged. Thrombus formation was moderate (++), and para-aortic hemorrhage was mild (+). Histologically, specimen F2 revealed a longitudinal medial split with hemorrhage and thrombus. The surrounding media displayed disrupted fiber architecture, and section F3 revealed a circular media defect consistent with the iatrogenically introduced wire. Adjacent areas showed mild fiber dehiscence and localized bleeding.

Specimen 22-24M (animal 1.1/24), part of the control group, exhibited mild intimal disruption (+) and a moderate medial tear (++++), with a minimal adventitial tear (+). Vessel wall hemorrhage was absent, but focal rupture of both elastic and muscle fibers was noted. A small amount of thrombus/fibrin was seen (+), and para-aortic hemorrhage was also present (+). While most of the vessel wall appeared unremarkable, section M2 revealed a small luminal hemorrhage with an early-stage fibrin clot. Section M3 showed focal media and adventitial splitting with minor adventitial hemorrhage and para-aortic lymph node involvement. M4 presented minor medial dissection and fiber dehiscence. In contrast, M6 showed no significant damage.

In summary, the histopathological findings confirm the successful and reproducible generation of an acute Stanford type A aortic dissection in the swine model. The induced lesions replicate the pathoanatomic features of human dissection, including characteristic entry tears, medial delamination, intramural hematoma, and thrombus formation. Importantly, distal wall segments beyond the dissection remained structurally intact, ensuring feasibility for longitudinal monitoring and biomarker evaluation. These results provide a robust histological basis to validate the model for further translational research, including studies on pathophysiology, diagnostics, and targeted therapies.

Overall, this model yielded consistent, reproducible, and histopathologically valid dissections mimicking human Stanford type A pathology. The dissection extent and vascular changes mirrored those observed in clinical scenarios, validating the technique as a suitable platform for translational cardiovascular research.

## 4. Discussion

Stanford type A aortic dissection (TAAD) remains one of the most life-threatening cardiovascular emergencies, with mortality increasing by up to 1–2% per hour after symptom onset if untreated [2,3]. The unpredictable clinical onset of TAAD and ethical limitations in studying acute dissection in humans have highlighted the need for robust and reproducible animal models that closely reflect human pathophysiology. This underscores the critical role of large-animal models, particularly in swine, which offer comparable vessel size, hemodynamics, and thoracic anatomy [11,14,15,16,17,18,19]. The model described in this study introduces a novel hybrid approach combining surgical access and endovascular manipulation to create standardized dissections in the ascending aorta, closely mimicking human TAAD in morphology, pathology, and disease behavior [5,6,7,8,9,10,11,12,13].

### 4.1. Histopathological and Macroscopic Validation

Macroscopic and microscopic evaluation of the explanted aortic specimens confirmed hallmark features of human TAAD, including intimal tears, medial delamination, thrombosed and patent false lumens, and para-aortic hematomas. Focal and segmental dissections extending through the intima into the media, and in some cases reaching the adventitia, were consistently produced. Histological staining (HE, EvG, Ladewig) revealed elastic fiber fragmentation, smooth muscle cell loss, intramural hemorrhage, and fibrin deposition—indicative of acute vessel wall trauma. In 5 of 6 animals, the lesions were reproducible, and distal aortic integrity was preserved, providing a model suitable for longitudinal biomarker and intervention studies. These findings align with the histopathological observations in acute human TAAD specimens, as reported by Halushka et al. (2016) and Leone et al. (2018), where medial degeneration, thrombus formation, and localized adventitial rupture were prominent features [20,21]. Our model’s ability to recreate such features in a time-controlled experimental setting is a significant advancement. Moreover, the macroscopic length of the induced dissections ranged from 1.0 to 5.8 cm. This variability reflects both anatomical differences between individual animals and the intentionally limited iatrogenic injury to the ascending aorta and proximal arch. While this extent is smaller than that of classical Stanford type A dissections in humans, it reproduces the key morphological and histopathological hallmarks of the disease. Future long-term studies will employ contrast-enhanced CT angiography and cardiac MRI to quantify dissection propagation and enable direct comparison with clinical imaging findings.

### 4.2. Comparison with Existing Large-Animal Models

Fujii et al. (2000) created an early swine model of aortic dissection by mechanically traumatizing the thoracic aorta using cross-clamping and high-pressure saline injection [6]. While some specimens showed dissection-like morphology, the technique suffered from unpredictability, high perioperative mortality, and limited applicability to TAAD. Importantly, dissections occurred in the descending thoracic aorta, anatomically analogous to Stanford type B disease. Moreover, the resulting tissue damage often extended beyond the medial layer, complicating interpretation. In contrast, our model introduces dissection in the ascending aorta, the hallmark of Stanford type A, and utilizes an iatrogenic guidewire injury, limiting excessive wall destruction. This enables better control over dissection location, depth, and extent.

Okuno et al. (2012) reported a reproducible model of type B dissection by endovascularly advancing a stiff guidewire and inflating a balloon catheter in the descending aorta. While elegant and minimally invasive, their model failed to recreate the critical features of TAAD, such as extension into the aortic root or arch and involvement of cerebral and coronary vessels [9].

Peng et al. (2023) recently evaluated a complete endovascular repair system for Stanford type A dissection in pigs [13]. They induced dissection using a high-pressure balloon in the ascending aorta, followed by stent graft deployment. While successful in demonstrating the feasibility of repair, the dissection itself was limited to short segments and lacked histopathological characterization.

Our model bridges the gap by providing surgical exposure for direct verification of injury and integrating endovascular components to induce consistent dissection while preserving natural tissue behavior. This hybrid methodology allows for both controlled lesion creation and real-time assessment, combining the strengths of both approaches.

### 4.3. Advantages of the Current Model

The porcine model presented in this study provides a range of advantages that make it particularly suited for translational research in Stanford type A aortic dissection. Anatomically, the swine ascending aorta closely resembles that of humans in terms of diameter, wall structure, and branching pattern. This similarity enables realistic testing of surgical procedures and endovascular devices under conditions that are directly translatable to clinical practice. The model allows precise control over the location and extent of the dissection, as the intimal tear is induced using prepositioned guidewires under direct surgical visualization. This can facilitate targeted creation of entry points that replicate clinical tear zones, such as those involving the sinotubular junction or proximal arch.

Importantly, the model demonstrated consistent histopathological features across animals, including intimal flaps, medial delamination, and fibrin-rich thrombus formation. This reproducibility provides a reliable basis for comparative and longitudinal studies. Hemodynamic stability was maintained in the majority of animals for six hours or more, which is critical for real-time biomarker sampling, physiological monitoring, and evaluation of acute therapeutic interventions. Furthermore, the model offers considerable flexibility, allowing researchers to adjust dissection parameters such as location (e.g., involvement of the aortic root or arch), extent, and the status of the false lumen (patent versus thrombosed). These attributes make the model well-suited for testing a wide spectrum of surgical, endovascular, and pharmacologic strategies.

Small-animal studies have used elastase or collagenase to enzymatically weaken the aortic wall, mimicking medial degeneration seen in Marfan syndrome or cystic medial necrosis. However, these models often require weeks to develop aortic disease, and dissection is not reliably achieved [19]. Furthermore, rodent anatomy and aortic biomechanics differ substantially from humans, limiting their translational utility.

Our model, in contrast, allows for acute induction of dissection within minutes and provides large tissue volumes for advanced imaging, histology, and molecular analyses. This is especially important for studies targeting temporal dynamics of inflammation, biomarker release, or mechanotransduction in the dissected vessel wall.

### 4.4. Clinical and Translational Implications

The translational value of the model lies in its capacity to reproduce clinically relevant challenges in the management of acute TAAD [22,23,24,25,26,27,28]. It provides a high-fidelity environment in which cardiac surgeons can refine complex aortic procedures, such as total arch replacement or hybrid repairs, under conditions that closely approximate human pathology. The anatomical congruence with human aortic dimensions permits the evaluation of next-generation graft materials, endovascular stent designs, and branched device prototypes within a realistic surgical setting. In addition, the controlled timing and progression of the dissection allow systematic blood sampling at defined intervals, facilitating the discovery and validation of circulating biomarkers associated with different pathological stages. From a pathophysiological standpoint, the model enables mechanistic investigations into early events in dissection biology, such as endothelial dysfunction, extracellular matrix degradation, smooth muscle cell apoptosis, and coagulation cascade activation. These insights are difficult to obtain in the clinical setting due to the unpredictability of disease onset. Ultimately, the model serves as a bridge between bench and bedside by enabling studies that integrate surgical practice, molecular biology, imaging, and therapeutic development, all within a reproducible and ethically manageable framework.

### 4.5. Future Directions

Future refinements of this model aim to increase its clinical realism and utility for long-term studies [22,23,24,25,26,27,28]. One of the main objectives is to extend the follow-up period beyond the acute phase to investigate chronic aortic remodeling, aneurysm formation, and the risk of re-dissection. Planned studies with survival endpoints at 7 and 14 days will incorporate imaging modalities such as Echocardiography, contrast-enhanced CT angiography and cardiac magnetic resonance imaging (MRI) to monitor anatomical changes over time and assess vessel wall healing. To further simulate the clinical scenario, pre-existing hypertension will be pharmacologically induced in selected animals. This will allow investigation of the role of elevated hemodynamic stress in dissection propagation and false lumen stability. Furthermore, pharmacologic induction of systemic hypertension will be standardized in selected experiments to evaluate its influence on proximal pressure gradients and the extent of dissection propagation in vivo. In parallel, integration of high-resolution imaging techniques will facilitate non-invasive assessment of dissection progression and enable comparison with human radiological findings.

Advanced molecular profiling approaches are also being explored. Tissue-specific sampling using laser-capture microdissection, combined with single-cell RNA sequencing, will enable in-depth transcriptomic analysis of different regions of the dissected aorta. This will provide insight into the cellular heterogeneity and molecular signaling pathways that drive the acute and subacute phases of dissection. Ultimately, these enhancements aim to establish the model as a versatile preclinical platform not only for mechanistic research but also for the development and testing of diagnostics and emergency treatment strategies.

## 5. Limitations

Despite the promising results and successful establishment of a reproducible acute Stanford type A dissection model in swine, several limitations must be acknowledged. First, the model represents an acute, non-survival setup with a 12 h observation period. While this timeframe allows for the evaluation of immediate pathophysiological and biomarker responses, it precludes insights into chronic remodeling processes, long-term survival, or delayed complications such as false lumen thrombosis, aneurysm formation, or re-dissection.

Second, due to the unavailability of advanced imaging modalities, such as Echocardiography, CT, or MRI, during the procedures, the evaluation of dissection extent and anatomical accuracy relied solely on macroscopic and histological assessments. Although histopathological validation confirmed the presence of hallmark dissection features, the absence of in vivo imaging limits direct correlation with clinical diagnostic modalities and dynamic visualization of dissection progression.

Third, although the model successfully mimics key anatomic and histological aspects of human TAAD, it does not fully recapitulate the hemodynamic and genetic heterogeneity observed in patients, such as those with Marfan syndrome, bicuspid aortic valve, or chronic hypertension. The artificial induction of the intimal tear and false lumen via surgical and endovascular means may also differ biomechanically from spontaneous dissection, potentially influencing tissue response or therapeutic performance. Additionally, the dissections were created in macroscopically and histologically healthy aortas without pre-existing medial degeneration. This design ensured technical reproducibility but limits comparability to spontaneous human dissections arising from chronic wall pathology. To address this, future studies will include protocols with a weakened aorta or pharmacologic induction of hypertension to simulate degenerative wall changes and enhance translational relevance.

Finally, the relatively small sample size (n = 6) limits statistical power and generalizability. Future studies should include larger cohorts and extended follow-up periods to enhance translational applicability and allow robust evaluation of interventional strategies, imaging techniques, and biomarker profiles.

## Figures and Tables

**Table 1:** Study design.

			Investigation			
Nr.	Animal	Experimental Setup	Baseline T1		Dissection T2	12 h-Follow-Up T3-T14
1	1/24	Narcosis and intubation Arterial and venous cannulation				
2	2/24					
3	3/24					
4	4/24	3. Sternotomie				
5	5/24	4. Dissection procedure				
6	6/24	5. Examination				
7	1.1/24	Control group				
8	1.2/24	Control group				

**Table 2:** Examination Numbers.

No.	Animal No.	Procedure	Pathlogic No.
1	1/24	Aorta ascendens	22-24A
2	2/24	Aorta ascendens	22-24B
3	3/24	Aorta ascendens	22-24C +
4	4/24	Aorta ascendens	22-24D
5	5/24	Aorta ascendens	22-24E +
6	6/24	Aorta ascendens	22-24F
7	1.1/24	Controll	22-24M +
8	1.2/24	Controll	22-24N

+—additional digital microscopic examination.

**Table 3:** Results of Microscopic Examination.

No.	Animal No.	Pathlogic No.	Localization Dissection	Proof of Dissection
1	1/24	22-24A	Aorta ascendens	Dissection of 1.0 cm length
2	2/24	22-24B	Aorta ascendens	Local Dissection, Perforation
3	3/24	22-24C +	Aorta ascendens	Dissection of 1.0 cm length; Digitalmicroscopy evaluation
4	4/24	22-24D	Aorta ascendens	Dissection of 1.5 cm length
5	5/24	22-24E +	Aorta ascendens	Dissection of 1.0 cm length, Digital Microscopy Evaluation
6	6/24	22-24F	Aorta ascendens	Dissection of 5.8 cm length
7	1.1/24	22-24M +	Controll aorta	No Dissection
8	1.2/24	22-24N	Controll aorta	No Dissection

## Data Availability

The data supporting this study’s findings are available from the corresponding author (Ezin Deniz) upon reasonable request.

## Supplementary Materials

The following supporting information can be downloaded at: https://www.mdpi.com/article/10.3390/jcdd12120496/s1, Figure S1: Microscopic examination of the explanted aortic specimens from animal 1/24 (Pathologic No. 22-24A2); Figure S2: Microscopic examination of the explanted aortic specimens from animal 3/24 (Pathologic No. 22-24C+); Figure S3: Microscopic examination of the explanted aortic specimens from animal 4/24 (Pathologic No. 22-24D); Figure S4: Microscopic examination of the explanted aortic specimens from animal 6/24 (Pathologic No. 22-24F); Table S1: Semiquantitative histological analysis of the explanted aortic specimens.
