# Supplementary material for: Development and Validation of an Acute Large Animal Model for Type A Aortic Dissection
Source: J Cardiovasc Dev Dis. 2025 Dec 16;12(12):496. doi: 10.3390/jcdd12120496 (PMC12733893; doi:10.3390/jcdd12120496)
Supplement: Supplementary file 1 [file jcdd-12-00496-s001.zip › jcdd-3912003-supplementary.pdf]

Supplements

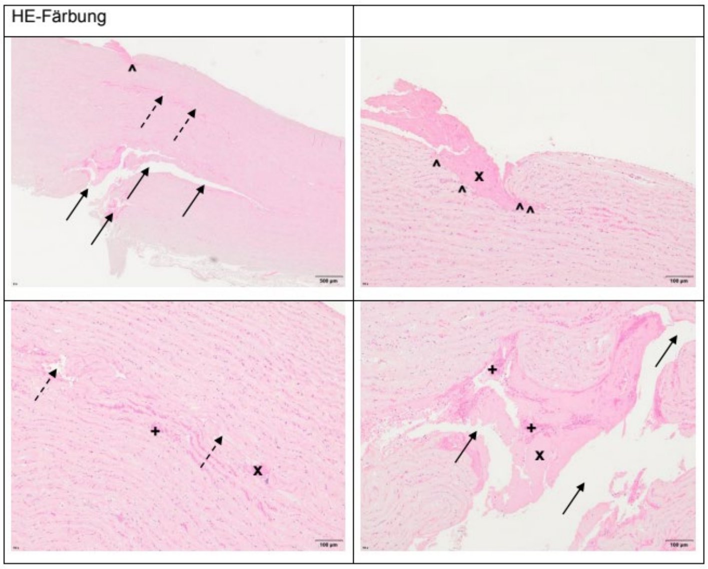

Figure S1: Microscopic examination of the explanted aortic specimens from animal 1/24 (Pathologic No. 22-24A2). In the overview, magnification, and detailed views, a lesion (arrow) extending through the media and reaching the adventitia can be seen. In addition, another tear in the intima (^) and media (dashed arrow) is visible, with focal indentations (+) and fibre bundle separations (x) present. (Upper row: 22-24A2_02; H&E stain, 20×; 22-24A2_15; H&E stain, 100×. Lower row: 22-24A2_12; H&E stain, 100×; 22-24A2_07; H&E stain, 100×.)

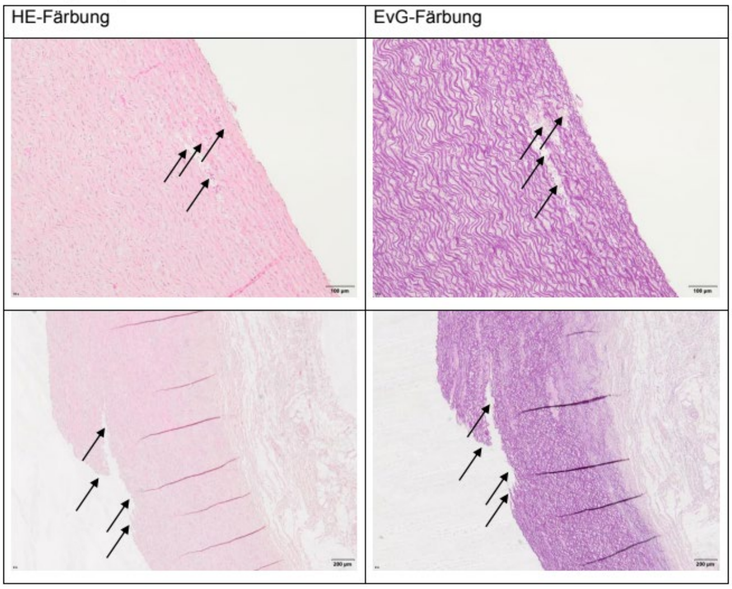

Figure S2: Microscopic examination of the explanted aortic specimens from animal 3/24 (Pathologic No. 22-24C+). In section 22-24C5, a focal hemorrhage with rupture of elastic fibers (upper row, arrows) as well as a tear in the intima and adjacent media (lower row, arrows) is visible. (Upper row: 22-24C5_06; H&E staining, 100x; 22-24C5_14; EvG staining, 100x. Lower row: 22-24C5_07; H&E staining, 40x; 22-24C5_14; EvG staining, 40x.)

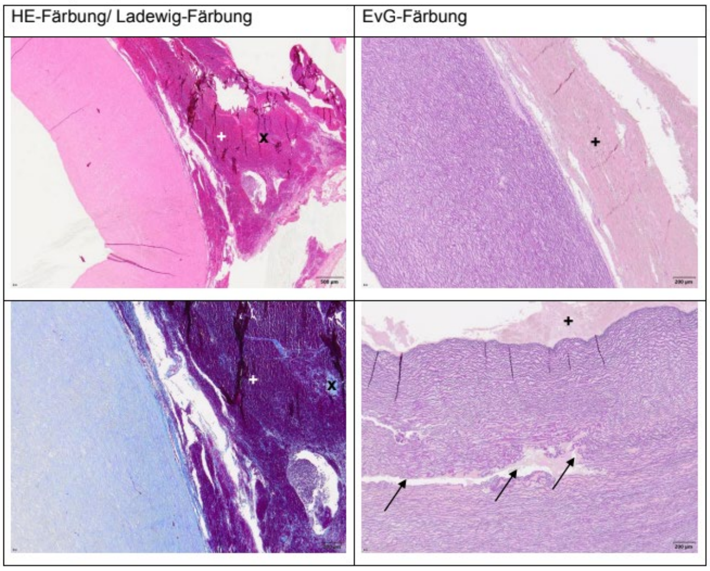

Figure S3: Microscopic examination of the explanted aortic specimens from animal 4/24 (Pathologic No. 22-24D). In section 22-24D2, a para-aortic hemorrhage (+) into the soft tissue is visible, with focal fibrin formation (x). Specimen 22-24D3 shows focal tearing of the media (arrows). (Upper row: 22-24D2_05; HE staining, 20x; 22-24D2_13; EvG staining, 40x. Lower row: 22-24D2_16; Ladewig staining, 40x; 22-24D3_08; EvG staining, 40x.)

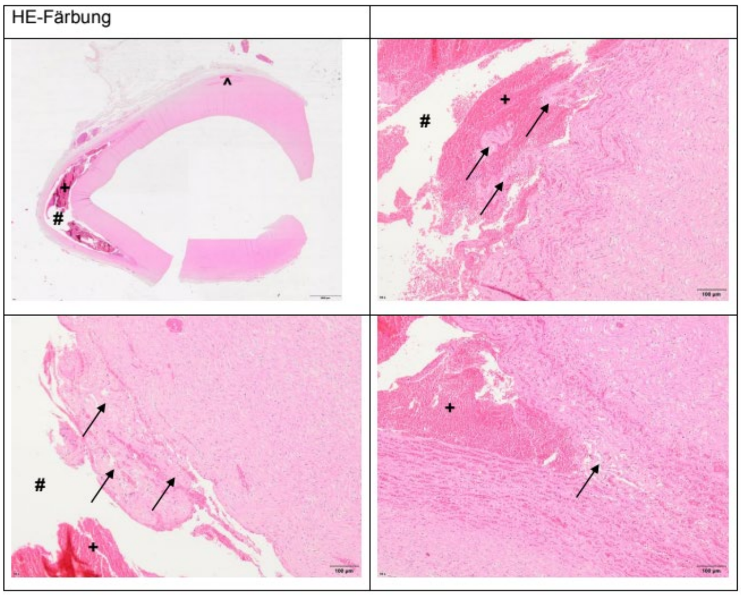

Figure S4: Microscopic examination of the explanted aortic specimens from animal 6/24 (Pathologic No. 22-24F). In section 22-24F2, a small tissue defect (^) is visible. Additionally, there is an extended splitting (#) in the media with cavity formation (+) and hemorrhage. Detached fibers of the media (arrows) and fragmented, partially rolled media components (arrows) can be observed. (Upper row: 22-24F2_97; HE staining, 12.5x; 22-24F2_21; HE staining, 100x. Lower row: 22-24F2_19; HE staining, 100x; 22-24F2_16; HE staining, 100x.)

Specimen	Intimal Tear	Medial Tear	Adventitial Tear	Vessel Wall Hemorrhage	Elastic Fibers	Muscle Fibers	Thrombus/Fibrin	Para-aortic Hemorrhage	Remarks
22-24A2 (1/24)	+	+++++	++	+	+++++ (rolled & ruptured)	+++++ (shortened & ruptured)	Fibrin deposition	-	Microscopically, a vessel wall section of the aorta is seen. While the wall structure appears mostly regular, there is a lesion extending from the media to the adventitia, with tearing and disruption of fiber alignment. Hemorrhages and fibrin deposits are evident. Surrounding fibers are interrupted and rolled at the edges. Luminal tears and focal hemorrhages with fibrin deposition are also visible.
22-24C4-6 (3/24)	+	+	-	(+)-+	Focal rupture, dehiscence	+ Focal rupture	++ (C6)	-	The cross-sections through the lesion (22-24C1–C3) became available after digital microscopic analysis. In 22-24C4, the vessel wall appears largely intact, aside from some preparation-related defects. In 22-24C5, focal hemorrhage with dehiscence and fiber rupture is evident. A tear through the intima and adjacent media is noted. 22-24C6 shows thrombus formation, fitting the profile of an initial clot.
22-24D (4/24)	-	++	++	++	+++++ (focal rupture, partially rolled, dehiscent, fiber accumulation)	+++++ (shortened & ruptured)	+++ (D1, D3–D6)	+++	In the tissue sections, the thrombus is visible in the lumen. The vessel wall shows minor para-aortic hemorrhages. In adjacent sections, especially 22-24D2 and D3, clear intimal and adventitial tears are seen, with dehiscence and fiber accumulation. Specimen 22-24D4 shows an unremarkable vessel wall structure. Specimens D1 and D2 indicate thrombus of coagulation type; D3–D6 show thrombi of mixed type.
22-24F (6/24)		+++		++	Rolled, fragmented, and ruptured	Shortened, fragmented, and ruptured	++	+	Sample 22-24F1 shows paraaortic bleeding into adjacent soft tissues. Tissue section 22-24F2 reveals a small defect and an extensive splitting in the media, with intramural hemorrhage and thrombus formation, including focal fibrin. Media fragments and tears with rolling of adjacent elastic fibers are present. Section 22-24F3 shows a circular defect in the media, traceable to the path of the iatrogenically introduced wire. Adjacent to this, there is a slight dehiscence in the fiber arrangement and focal bleeding.
22-24M (1.1/24)	+	++++	(+)	-	Focal rupture	Focal rupture	+	+	Samples 22-24M1 and 22-24M2 show an unremarkable vessel wall. Sample 22-24M2 includes a small luminal hemorrhage with an initial fibrin clot. Section 22-24M3 shows focal splitting in the media and adventitia with minor adventitial bleeding and paraaortic lymph nodes. In 22-24M4, focal media dissection and fiber dehiscence occur. Sample 22-24M6 shows no defect or adventitial tear. Focal splitting in 22-24M2 suggests luminal hemorrhage with fibrin deposition and paraaortic lymph node with sinus histiocytosis.

Table S1: Semiquantitative histological analysis of the explanted aortic specimens. (+)=minimal; (++)=mild; (+++)=moderat; (++++)=Pronounced/extensive; (+++++)=extremely pronounced/extensive
